# Safety design guidelines for clinician–AI interaction in computer-aided diagnosis systems using system-theoretic framework with explainability validation

**DOI:** 10.1038/s41598-026-64180-w

**Published:** 2026-07-29

**Authors:** Yuki Hagiwara, Katherine Fitch, Mario Trapp

**Affiliations:** 1 Fraunhofer Institute for Cognitive Systems IKS, Garching bei München, Germany; 2 Engineering Resilient Cognitive Systems, Technical University of Munich, Garching bei München, Germany

**Keywords:** Computer-aided diagnosis, Clinician-AI interaction, Explainable AI, Human-centered AI, Systems-theoretic process analysis, Trust calibration, Computational biology and bioinformatics, Engineering, Health care, Mathematics and computing

## Abstract

Ensuring the safety of Artificial Intelligence-enabled Computer-Aided Diagnosis systems is critical because diagnostic errors can have serious consequences for patient care. However, existing regulatory and risk management frameworks often do not sufficiently address the complex socio-technical interactions between clinicians and Artificial Intelligent systems, leaving key human-centered safety challenges underexplored. This paper presents a systematic and human-centered approach to deriving safety design guidelines for clinician-Artificial Intelligence interaction in Computer-Aided Diagnosis systems using System-Theoretic Process Analysis. Through this analysis, we identify critical hazards associated with clinician–Artificial Intelligence collaboration, including automation bias on system recommendations, and misinterpretation of explanations. Based on the identified unsafe control actions, we formulate a set of actionable and traceable safety design guidelines that promote transparency, coherent explanations, and calibrated trust in Artificial Intelligence-assisted decision-making. To bridge safety analysis and system design, the proposed guidelines are operationalized within a Computer-Aided Diagnosis interaction framework. The framework includes a safety-oriented Graphical User Interface that integrates multiple explanation methods and interactive mechanisms to promote clinician engagement. Furthermore, we introduce a safety-oriented evaluation approach that uses consistency across multiple explanation methods as a quantitative indicator of potentially unreliable or ambiguous explanations. By linking System-Theoretic Process Analysis, interaction design, and explainability evaluation, this work provides a unified and reusable framework for improving the safety and reliability of Artificial Intelligence-driven Computer-Aided Diagnosis systems.

## Introduction

Artificial Intelligence (AI)-enabled Computer-Aided Diagnosis (CADx) systems are increasingly adopted to support clinicians in medical decision-making across domains such as radiology, pathology, and dermatology. By analyzing medical data and assisting in distinguishing between healthy and diseased cases, these systems can enhance diagnostic accuracy and efficiency. However, as diagnostic decisions directly affect patient outcomes, CADx systems are inherently high-risk, safety-critical tools, where errors may lead to severe harm.

The integration of AI into CADx systems introduces safety challenges beyond those addressed in traditional medical devices. Modern AI models, particularly those based on Deep Learning (DL), often operate as “black boxes”, limiting transparency into their decision-making processes. This opacity can lead to clinician uncertainty, misinterpretation of AI outputs, and inappropriate reliance on system recommendations. Moreover, clinician-AI interaction introduces socio-technical risks such as automation bias and increased cognitive workload during verification. These challenges highlight the need for approaches that consider not only model performance but also how AI systems are interpreted and used in clinical workflows.

Traditional safety frameworks such as DIN EN ISO 14971:2022-04 (Medical Devices - Application of Risk Management to Medical Devices)^1^ primarily focus on risks that directly lead to patient harm. While effective for conventional medical devices, they do not fully capture the dynamic and interaction-driven risks introduced by AI-enabled systems. This is particularly evident in clinician-AI collaboration, where risks such as misinterpretation of explanations and inappropriate trust calibration arise.

Current medical device regulations such as the EU 2017/745 Medical Device Regulation (MDR)^2^ and 2017/746 In Vitro Diagnostic Medical Devices Regulation (IVDR)^3^ provide general safety requirements for medical devices but were developed before the widespread adoption of AI. As a result, they provide limited guidance for managing risks arising from clinician-AI interaction. Likewise, emerging initiatives including the American Association of Physicists in Medicine (AAPM)^4^ best practices, FUTURE-AI consortium^5^ principles, and the AAMI/BSI^6^ guidance primarily focus on robustness, fairness, and data quality instead of interaction-level safety concerns, which includes explanation interpretation and trust calibration.

To bridge this gap, this paper presents a human-centered and systematic approach to analyzing and mitigating risks in clinician-AI interaction for CADx systems. We employ System-Theoretic Process Analysis (STPA), a top-down safety engineering method that models unsafe interactions within complex socio-technical systems^7^ to identify hazards and loss scenarios arising from both technical and human factors. Based on this analysis, we derive a set of actionable and traceable safety design guidelines that support transparency, coherence of explanations, and calibrated trust in clinical decision-making. Moreover, to bridge safety analysis and system design, these guidelines are operationalized within a CADx interaction framework, demonstrating how system-theoretic safety principles can inform the design of safe and interpretable interfaces.

In addition, we introduce a safety-oriented evaluation approach that leverages consistency across multiple Explainable AI (XAI) methods as a quantitative signal for identifying potentially unreliable or ambiguous explanations. By analyzing agreement and disagreement between XAI methods, we provide a mechanism for detecting scenarios where clinician misinterpretation or over-reliance may occur. Unlike conventional evaluations that assess XAI methods in isolation, this work focuses on interaction-level safety in clinician-AI collaboration. Our contributions are threefold: (1) the application of STPA to clinician-AI interaction with explainability mechanisms, (2) the derivation and operationalization of safety design guidelines within a CADx interaction framework, and (3) a safety-oriented evaluation approach that treats explanation consistency as a quantitative signal of potential interaction risks.

This work is guided by the following research question: *How can system-theoretic safety analysis be used to systematically derive actionable and traceable safety design guidelines for clinician-AI interaction in CADx systems and how can these guidelines be supported through measurable indicators of explanation consistency and reliability?*

The contributions of this work are as follows:

- A human-centered safety analysis framework for CADx systems that explicitly models clinician-AI interaction, enabling the identification of socio-technical hazards that are not captured by traditional risk analysis methods.
- An extension of STPA to AI-driven CADx systems, incorporating explainability and human decision-making factors to systematically analyze risks arising from clinician-AI collaboration.
- A set of actionable and traceable safety design guidelines derived from unsafe control actions, translating system-level safety constraints into interaction and interface design principles that support transparency, coherence of explanations, and calibrated clinician trust.
- A safety-informed interaction framework that operationalizes these guidelines within a CADx interface, including a GUI design and adaptive feedback mechanisms to mitigate identified risks during clinician-AI interaction.
- A safety-oriented empirical validation demonstrating that consistency across multiple XAI methods can be used as a quantitative signal to detect potential risk scenarios, thereby providing a practical and measurable mechanism for evaluating and supporting the proposed safety design guidelines.

The remainder of this paper is structured as follows. Section Background and related work provides an overview of CADx systems and reviews existing safety and human-AI interaction frameworks. Section System-theoretic process analysis describes the STPA methodology and its application to CADx systems, detailing the identified hazards, unsafe control actions, and system level safety constraints in clinician-AI interaction. Section Guidelines presents the derived safety design guidelines for clinician-AI interaction, abstracted from STPA. Section Evaluation and validation presents the empirical evaluation, including consistency analysis, comparative studies, and scenario-based validation. Section Discussion discusses the implications of the results and the traceability of the proposed guidelines for safe clinician-AI interaction. Finally, Conclusion and future work section concludes the paper with directions for future work on safety assurance in AI-driven diagnostic systems.

## Background and related work

### Computer-aided diagnosis system

There is extensive literature on both the employment of traditional Machine Learning (ML) and DL techniques to develop CADx systems in different medical applications. Chen et al. ^8^ employed traditional ML techniques to automatically detect and grade gliomas. Gao et al. ^9^ compared traditional ML and DL techniques utilized in breast imaging and emphasized how DL techniques have potential to surpass traditional CADx systems in mammography. Lee et al. ^10^ provided an extensive review on the introduction of DL in radiology applications from the perspectives of radiologists. They highlighted the shift towards DL in radiology applications and mentioned the various challenges of bringing this technology in radiology. Guetari et al. ^11^ presented a comprehensive review on the different types of traditional ML and DL algorithms and common metrics used in CADx systems such as feature extractor techniques, classifiers, etc. Yoon et al. ^12^ proposed a high performing CADx system to diagnose retinal diseases. However, these studies mainly focused on developing novel algorithms to achieve high-performing CADx systems.”

### Overview of current safety standards for computer-aided diagnosis systems

Numerous standards exist to regulate health AI software, including ISO 13485:2016 (Medical Devices—Quality Management Systems)^13^, IEC 62304:2006 (Medical Device Software – Software Lifecycle Processes)^14^, FDA guidelines, and EU MDR for medical devices. These standards provide a foundation for ensuring safety and quality in medical devices, but they do not fully address the unique complexities of AI technologies, particularly the advanced AI techniques like DL that are increasingly being applied in healthcare.

Recent AI-specific guidelines such as ISO/IEC TR 24028:2020 (Information Technology – Artificial Intelligence—Overview of trustworthiness in Artificial Intelligence)^15^, ISO/IEC 42001:2023 (Information Technology – Artificial Intelligence – Management System)^16^ and ISO/IEC 23053:2022 (Framework for Artificial Intelligence Systems Using Machine Learning)^17^ provide additional guidance on key aspects such as data management, model development, risk management, and product life cycle for AI. However, these standards remain general and are not tailored to the specific demands and safety-critical nature of healthcare applications, especially for CADx systems.

While these standards offer useful foundations for AI safety, they do not comprehensively cover AI safety across the entire life cycle of CADx systems. Gaps remain in areas such as the interaction of AI models and clinicians, where issues like automation bias on AI decisions could lead to unsafe outcomes. Ensuring robustness in such interactions is critical, as failures within the clinician-AI interaction whether in terms of functional insufficiency in the AI or human misunderstanding can impact diagnostic accuracy and patient safety.

### Human-AI interaction in computer-aided diagnosis systems

The integration of AI into CADx systems does not eliminate the need for human oversight, rather it creates a new dynamic between human healthcare professionals and AI outputs. While AI-based CADx systems offer powerful diagnostic capabilities, the final decision-making still lies with the healthcare professional. Current regulations require healthcare professional to verify the diagnoses provided by the CADx systems before communicating them to the patients. This interaction introduces unique safety challenges, as misinterpretation or over-reliance on AI-generated results could lead to diagnostic errors or patient harm. Factors such as the clarity of AI explanations, the level of trust the healthcare professionals have in AI recommendations, and their ability to assess these outputs can significantly influence diagnostic outcomes and patient safety. Misaligned or insufficient communication between the AI and human professionals can exacerbate these risks. Traditional safety analysis techniques struggle to fully address the complexities introduced by this human-AI interaction.

Recent research has explored the use of XAI techniques to improve transparency and support human-AI interaction in medical applications^18^. Methods such as saliency-based approaches, feature attribution techniques, and concept-based explanations have been proposed to make AI decisions more interpretable^19^.

However, several studies have highlighted that individual XAI methods can produce inconsistent or conflicting explanations, which may lead to confusion or misinterpretation by clinicians^20^. Furthermore, existing work mainly evaluates XAI methods in isolation, focusing on metrics such as faithfulness or localization accuracy, rather than assessing their role within interactive clinical decision-making workflows.

Although prior work has compared the behavior of different XAI methods and highlighted inconsistencies between explanations, these studies generally focused on evaluating explanation quality or method reliability. To the best of our knowledge, limited work has investigated explanation consistency as a safety-oriented signal within clinician-AI interaction or integrated such information into safety analysis and interface design.

This gap between XAI evaluation and interaction-level safety motivates the need for approaches that not only generate explanations but also evaluate and communicate potential disagreement to clinicians. Such mechanisms may provide additional signals for identifying situations where explanation reliability is uncertain.

### Safety analysis of computer-aided diagnosis systems

There are typically two main approaches to assuring the safety of AI-enabled safety-critical systems. The first approach involves identifying safety-related properties specific to the AI function and demonstrating that these properties are sufficient to construct a compelling assurance argument or assurance case. Ashmore et al. ^21^ presented a general template, called the AMLAS framework, for a safety ML life cycle based on desired properties like generalizability, robustness, and interpretability. Similarly, Picardi et al. ^22^ developed an assurance case pattern for AI-based clinical diagnosis systems, aimed at justifying the use of these systems in clinical environment by systematically addressing the potential risk of misdiagnosis or harm. These approaches are useful in ensuring that the AI function itself is safe and trustworthy, but they do not fully address the complexities of clinician-AI interaction in diagnostic decision-making.

The second approach employs classical safety analysis techniques to first assess the risks of the system and then systematically derive safety requirements. Molloy and McDermid ^23^ employed an adaptation of the Hazard and Operability Analysis (HAZOP) technique for safety analysis and subsequently derived safety requirements in order to minimize the occurrence of the hazard and ensure the safety of the system. Similarly, Jia et al. ^24^ employed a HAZOP-based safety analysis technique, also known as SHARD, with Bayesian network structure learning to ensure patient safety. Habli et al. ^25^ developed a methodology called the Safety Modelling, Assurance and Reporting Toolset (SMART) to identify hazards consistently and facilitate clinically meaningful risk analysis to improve patient safety. However, these approaches still tend to focus on AI functional risks, without fully integrating how clinicians interact with AI outputs, how they trust or rely on these systems, and how human error might arise from these interactions. Moreover, limited work has evaluated how safety design decisions influence the behavior of XAI systems and their interaction with users. There remains a need for frameworks that not derive safety requirements but also validate their impact through measurable system-level outcomes.

**Table 1:** Comparison of existing safety approaches and their support for clinician-AI interaction. “Minimal/Moderate/Explicit” indicate increasing explicitness in modeling human–AI interaction.

Approach	Primary focus	Explicitness of human-AI interaction	Description
AMLAS	Assurance of machine learning properties	Minimal	Focuses on AI assurance properties (e.g., robustness, interpretability) rather than interaction dynamics.
HAZOP / SHARD	Hazard identification through guidewords	Moderate	Identifies hazards but does not explicitly model feedback loops, trust calibration, or explanation interpretation.
SMART	Clinical safety risk modeling	Moderate	Supports structured risk analysis but provides limited support for modeling clinician reasoning and AI explanations.
STPA	Control structure and unsafe interactions	Explicit	Explicitly models feedback, human decision-making, unsafe control actions, and socio-technical interactions.

While existing approaches such as AMLAS, SMART, and HAZOP-based safety analyses provide valuable support for AI assurance and hazard identification, they mainly focus on the safety of AI functions, system failures, or operational deviations (see Table 1 for the summary of different safety approaches). Little attention is given to how clinicians interpret, trust, and act upon AI outputs during diagnostic decision-making. As a result, important interaction-level hazards e.g., over-reliance on AI recommendations, misinterpretation of explanations, and inappropriate trust calibration may be insufficiently addressed.

This gap motivates the need for a safety analysis approach that explicitly models clinician-AI interaction as part of a socio-technical system. Such an approach should systematically identify foreseeable misuse, human errors, unsafe interactions, and feedback-related risks that could compromise diagnostic safety. In this work, we adopt STPA to address these challenges and derive safety design guidelines for clinician-AI interaction.

## System-theoretic process analysis

STPA is particularly suited for addressing system-level safety where both technical and human factors play a significant role. This method is effective in complex systems like the CADx system, where clinician interaction with AI systems is critical for patient outcomes. By focusing on clinician-AI interaction, STPA allows for the systematic identification of risks arising not only from technical failures but also from potential human misuse, over-reliance on, or misinterpretation of the CADx system’s outputs.

**Fig. 1:**
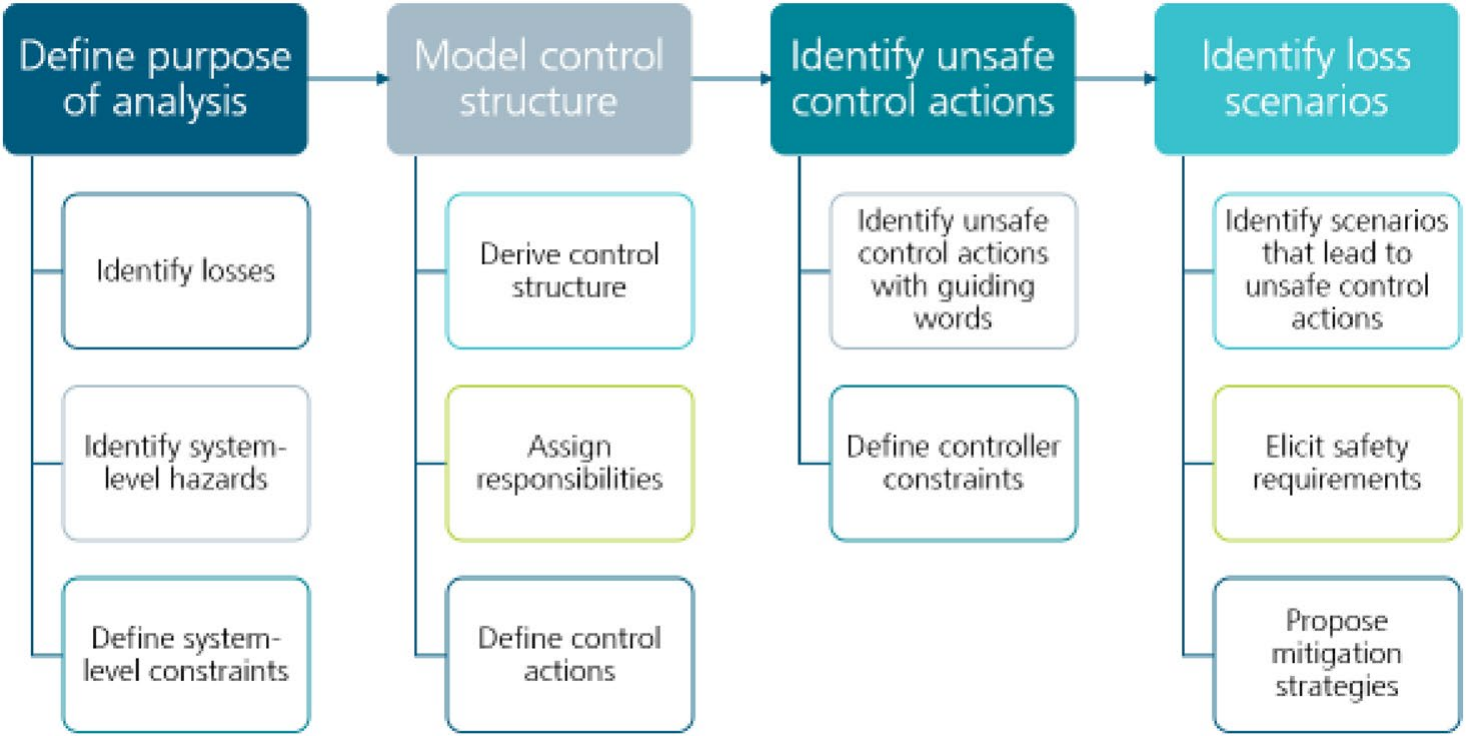
Four-step process of STPA.

Figure 1 illustrates the four-step process of STPA. The analysis begins by defining its purpose, including systems boundaries, unacceptable losses, and assumptions. Next, a control structure is modeled to represent how clinicians, the CADx system, the interface, and the clinical workflow interact through control and feedback loops. Unsafe Control Actions (UCAs) are then identified by examining how actions provided, not provided, or timed incorrectly by any controller (either by the CADx system or the clinician) could violate safety constraints. Finally, STPA identifies loss scenarios that explain why UCAs might occur, considering factors such as incomplete feedback, misleading explanations, automation bias, or mismatches between the clinician’s and the CADx system’s mental models. This approach allows a deeper, human-centered analysis of safety beyond traditional risk assessments.

In this use case, we examine the process of patient diagnosis and treatment delivery involving a CADx system, a clinician, and the patient. This process forms an integrated system in which clinician-AI plays a key role. Medical data is first acquired from the patient, then analyzed by the CADx system. The AI component in the CADx system generates diagnostic outputs based on the input data, which the clinician uses to inform treatment decisions. Importantly, the clinician’s interpretation and decision-making are influenced by the AI’s outputs, raising critical questions regarding clinician-AI interaction.

The focus of the STPA in this work is specifically on the interaction between the clinician and the CADx system. This includes safety concerns related to human behavior and decision-making in response to the CADx system outputs such as potential biases and communication breakdowns. Hence, AI-specific issues such as data quality and algorithmic failures, as well as other technical aspects including the data acquisition from the medical imaging equipment are excluded.

### Purpose of analysis

To start with the first step of STPA, we identify the losses, system-level hazards and their corresponding system-level constraints. Losses refer to undesirable outcomes that may result from failures in the interaction between the clinician and the CADx system. System-level hazards are conditions or states of the system, in combination with environmental factors, that can lead to a loss. System-level constraints are rules or safeguards to prevent the system (in this case, clinician-AI interaction) from entering these hazardous states.

The losses which we want to prevent in this work are as follows:

- L-1: Harm to the patient.
- L-2: Delayed or incorrect treatment decisions.
- L-3: Loss of clinician trust in CADx system.
- L-4: Inaccurate or unreliable diagnoses.

The system-level hazards which we want to prevent in this work are as follows:

- H-1: Clinician relies too heavily on CADx system (automation bias) [L-1, L-2, L-3].
- H-2: Algorithm aversion bias [L-1, L-2, L-4].
- H-3: Inconsistent or unclear explanations by CADx system [L-2, L-3, L-4].
- H-4: Communication breakdown between clinician and CADx system [L-1, L-2, L-3, L-4].
- H-5: Misalignment between clinician’s understanding and the output of CADx system [L-1, L-2, L-3, L-4].

The corresponding system-level constraints are defined as follows:

- SC-1: Clinician must not rely too heavily on CADx system (automation bias) [H-1].
- SC-2: Clinician must avoid algorithm aversion [H-2].
- SC-3: CADx system must provide consistent and clear explanations for its outputs [H-3].
- SC-4: Communication between clinician and CADx system must be seamless and clear [H-4].
- SC-5: The clinician’s understanding must align with the CADx system’s outputs [H-5].

### System control structure

**Fig. 2:**
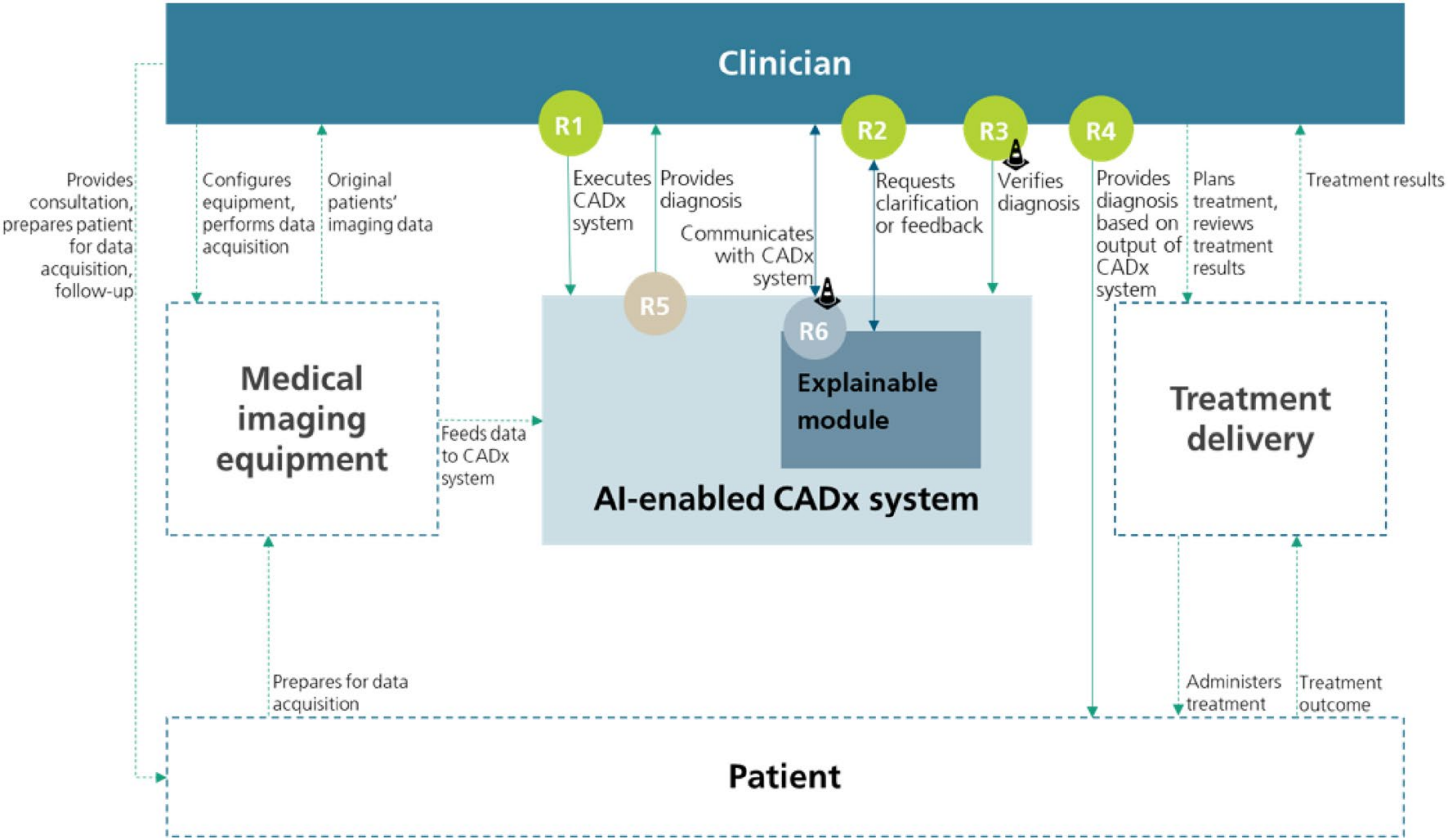
Control structure for CADx-enabled diagnosis centered on the clinician–AI interaction. The clinician, CADx system, and its explainable module form the primary information and feedback loops; arrows denote information/decision flow between these controllers. Medical imaging equipment provides diagnostic inputs; treatment delivery and the patient are shown for context only. Dotted elements indicate non-focus. Responsibilities R1–R6 are labeled.

The control structure is a representation of the functions that a system must perform and how the different components function together. The purpose of the control structure is to logically identify potential hazards through control actions and their origins in the system. Figure 2 portrays the control structure diagram illustrating patient diagnosis and treatment delivery process with the proposed clinician-AI interaction in a CADx system. Since the focus of this STPA is on the interaction between the clinician and the CADx system, the irrelevant components (patient, medical imaging equipment, and treatment delivery) are represented in dotted lines.

The clinician functions as the human controller, issuing control actions such as executing the CADx system, requesting clarification, verifying AI outputs, and making the final diagnostic decision (R1-R4). The CADx system acts as an automated controller that analyses patient data and produces diagnostic outputs (R5), while the embedded explainable module provides interpretability feedback to support clinician understanding (R6).

The arrows represent the control actions and feedback loops that STPA requires for identifying UCAs. The cones attached to “Clinician verifies diagnosis” (R3) and “Explainable module” (R6) denote points where process model inconsistencies may arise such as misleading explanations, missing information, or misinterpreted diagnostic cues which are triggers for UCAs in STPA.

The short role descriptions attached to each element clarify the functional responsibilities of each controller, which STPA uses to trace hazards back to flawed control, inadequate feedback, or incorrect assumptions in the controllers’ mental models.

The following control actions (in the context of analyzing the interactions between the clinician and the AI) are defined:

1. CADx system provides a diagnosis.
2. Clinician requests clarification from the CADx system.
3. Explainable module in the CADx system provides relevant explanations of the diagnosis.
4. Clinician verifies the diagnosis.
5. Clinician makes the final diagnostic decision based on the output and personal expertise.

### Unsafe control actions

The third step identifies potential causes via the defined control actions in System control structure section and a set of guiding words.

The guiding words according to STPA, are as follows:

- Not providing causes hazard: Control action is not executed when it should be.
- Providing causes hazard: Control action is executed incorrectly.
- Incorrect timing or order (Too early, too late): Control action is executed at the wrong time or in the wrong sequence.
- Stopped too soon or applied too long: Control action is not executed for the correct duration.

There are a total of 12 UCAs identified (see Table 4 in Appendix A for the full list of identified UCAs). They are seen as hazardous events in relation to its control structure diagram (see Fig. 2). Therefore, it is necessary to address them with appropriate safety argument or Controller Constraint (CC) (see Appendix B for the full list of CCs defined). These CCs specify the controller behavior that needs to be satisfied to prevent UCAs.

Representative UCAs highlight critical risks in clinician-AI interaction. For example, UCA-2 captures scenario where the CADx system provides incorrect or misleading diagnostic outputs, potentially reinforcing automation bias. UCA-8 reflects failures in XAI, where incomplete or unclear explanations may lead to misinterpretation of AI reasoning. From the clinician perspective, UCA-10 describes situations where diagnostic outputs are not adequately reviewed or verified, while UCA-12 captures cases of algorithm aversion where clinicians disregard AI recommendations entirely.

Although several identified UCAs are generally applicable to AI-assisted medical systems, a subset is specific to the incorporation of XAI into clinician-AI interaction. In particular, UCAs involving misleading or inconsistent explanations and failures to communicate uncertainty extend beyond conventional STPA analyses of medical devices by explicitly modeling explainability as part of the interaction loop.

While UCAs identify conditions under which system behavior becomes hazardous, they do not fully explain how these situations arise in practice. Therefore, the next step of the analysis focuses on identifying loss scenarios, which describe the underlying causal factors that can lead to these UCAs.

### Loss scenarios

Loss scenarios describe the causal factors that lead to UCAs and hazards. To derive the loss scenarios, we followed the standard STPA procedure by examining each identified UCA and tracing the potential causal factors that could lead to it. This includes analyzing how inadequate control, delayed, or missing feedback, flawed assumptions in the clinician’s or CADx system’s process models, or external disturbances could create conditions in which a UCA becomes possible. We systematically evaluated each relevant control action within the control structure and asked STPA guide words such as the following:

- Failures related to the controller: CADx system, clinician, explainable module
- Inadequate control algorithm: Flaws in diagnosis algorithms, explanation mechanisms
- Unsafe control input: Incorrect clinician inputs or misinterpreted feedback
- Inadequate process model: Misinterpretations, delays, lack of feedback in CADx system

For example, a loss scenario may arise when the CADx system provides an explanation that is technically correct but not aligned with the clinician’s mental model, leading to misinterpretation and potential diagnostic error. There are a total of 22 loss scenarios identified through this process, capturing a wide range of potential failures in clinician-AI interaction. The complete list of loss scenarios is provided in Appendix C.

From our analysis, we identify four primary areas: (1) algorithmic limitations, (2) system resource constraints, (3) GUI design gaps, and (4) clinicians’ trust. We prioritize addressing GUI design gaps and fostering clinicians’ trust, as these directly influence clinician-AI interaction. This categorization provides a structured view of how safety risks emerge across both technical and human dimensions of clinician-AI interaction. While no hazard analysis can guarantee absolute completeness, this structured, top-down reasoning process ensures that the loss scenarios cover the full space of human-AI interaction failures relevant to the CADx system workflow.

Moreover, from this analysis, recurring patterns are observed, particularly in relation to explanation clarity, system transparency, and the calibration of clinician trust. These patterns form the basis for the derivation of higher-level safety design guidelines which will be presented in Guidelines section.

## Guidelines

As a further step, we derive a set of actionable safety design guidelines for clinician-AI interaction in CADx systems based on the STPA approach presented in System-theoretic process analysis section . These guidelines are systematically abstracted from the identified UCAs and CCs, ensuring traceability between safety analysis and design recommendations. They address socio-technical challenges, including explanation clarity, trust calibration, and interaction design, and translate system-level safety constraints into practical interface and system design principles.

The safety design guidelines for clinician-AI interaction are as follows:

- G1: Ensure clarity and interpretability of AI explanations AI-generated explanations should be presented in a clear, structured, and clinically relevant manner to support accurate interpretation and reduce the risk of misunderstanding.
- G2: Communicate CADx system status and reliability transparently CADx systems should provide clear feedback on processing status and diagnostic confidence to support informed decision-making and prevent misinterpretation of system outputs.
- G3: Support interactive and on-demand explanation Clinicians should be able to request additional explanations or clarification through interactive interface elements, enabling deeper understanding when needed.
- G4: Promote calibrated trust and reflective decision-making System design should encourage appropriate reliance on AI by supporting critical evaluation, preventing automation bias, and discouraging algorithm aversion.
- G5: Ensure consistency and coherence across XAI outputs Outputs from multiple XAI modules should be aligned and presented coherently, with discrepancies clearly indicated to avoid confusion and loss of trust.
- G6: Minimize cognitive load and support efficient clinical workflow Interfaces should present information in a structured and non-overwhelming manner, integrating relevant patient data and explanations to support efficient and context-aware decision-making.

Together, these guidelines provide a structured and reusable framework for designing safe and effective human-AI interaction in medical diagnostic systems.

To reinforce clinician trust further, we aim to ensure alignment between the CADx system’s diagnostic outputs and the associated explanations provided by the XAI models. Consistency across these explainable elements is crucial, as any discrepancies could introduce doubt, potentially undermining clinicians’ confidence in the CADx system. The detailed mapping from Safety Goals (SGs), Safety Requirements (SRs), and Mitigation Strategies (MSs) to the proposed guidelines is provided in Appendix D.

### Operationalized in the computer-aided diagnosis interface

To demonstrate the practical applicability of the proposed safety design guidelines, we developed a working prototype of a safety-oriented CADx interaction framework. Rather than serving as a conceptual illustration alone, the prototype implements key interaction mechanisms derived from the STPA and provides a foundation for future empirical evaluation through clinician-centered user studies.

**Fig. 3:**
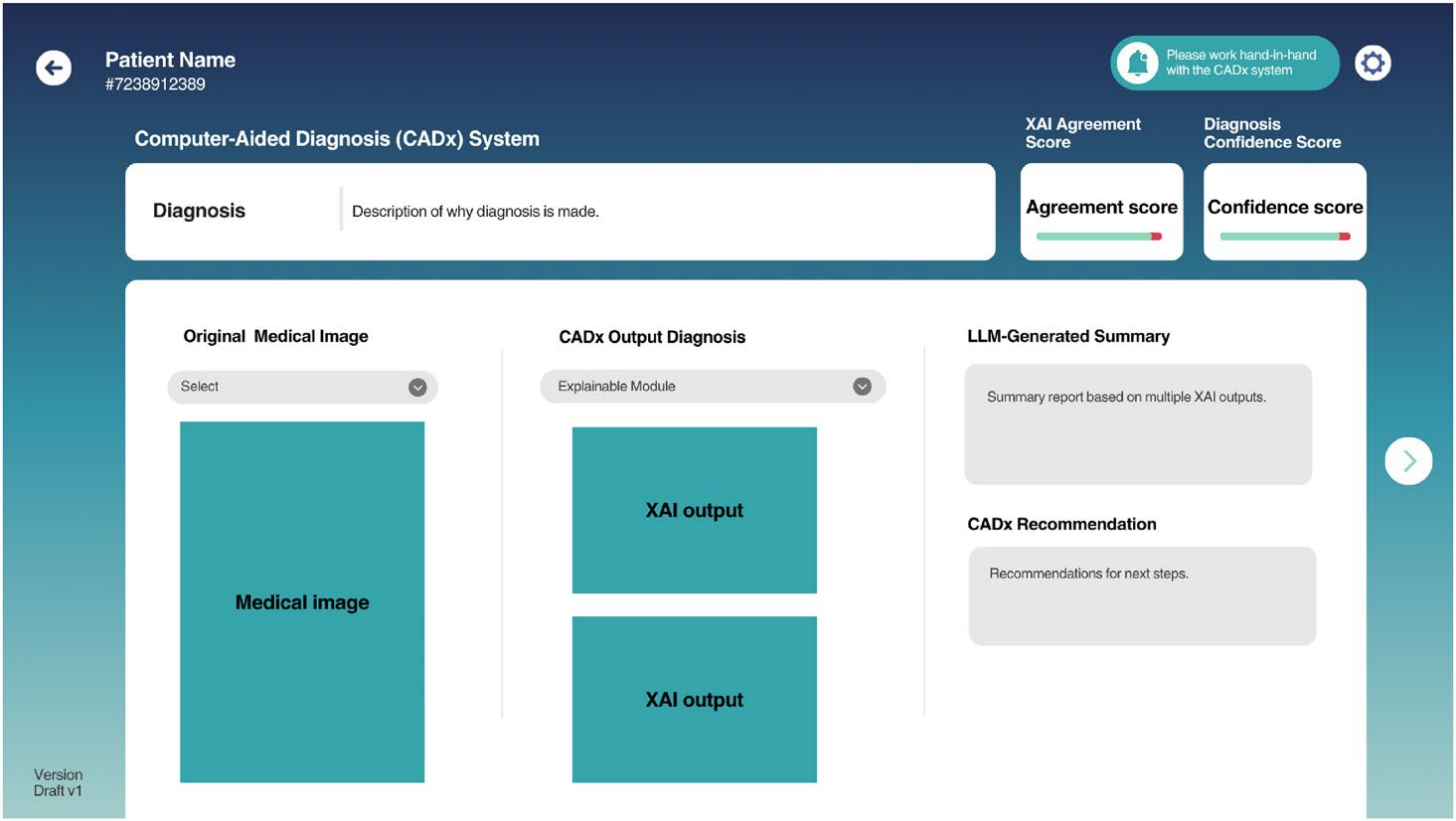
Proof-of-concept implementation of the proposed safety-oriented CADx interaction framework, illustrating how the derived design guidelines are operationalized within the user interface.

Figure 3 illustrates a proof-of-concept of the proposed CADx interface that operationalizes the derived safety design guidelines. The prototype serves as a proof-of-concept demonstrating how STPA-derived interaction requirements can be translated into concrete interface elements for clinician-AI collaboration. A formal usability evaluation is planned as future work. The design emphasizes clarity, transparency, and support for collaborative reasoning. A concise, LLM-generated summary assists clinicians in understanding the rationale behind the CADx system’s recommendation by synthesizing insights from multiple XAI modules. To further support interpretability and trust calibration, both an agreement score and a confidence score are presented. The agreement score reflects the extent of consistency among different XAI outputs, while the confidence score indicates how confident the CADx system is in the diagnosis made. Interactive features, such as prompts encouraging active engagement, help promote shared decision-making and reduce over-reliance on AI outputs. Additionally, accessible tooltips and training materials available via the “settings” icon help clinicians understand the CADx system’s intended use, operational boundaries, and limitations. XAI outputs are dynamically displayed according to selected explainability modules, and relevant patient details are integrated into the interface to support contextualized decision-making. Finally, the interface includes LLM-generated suggested next steps, bridging AI-driven insights with clinical reasoning.

## Evaluation and validation

This section evaluates the proposed safety-oriented clinician-AI interaction framework by examining if the derived design guidelines effectively address the risks identified from STPA. In contrast to conventional evaluations that focus solely on model performance or explanation quality, our evaluation is explicitly framed from a safety perspective. We also investigate if explanation consistency across multiple XAI methods can serve as a reliable indicator of potential hazards in clinician-AI interaction.

We hypothesize that disagreement between independent XAI methods reflects increased uncertainty or ambiguity in model reasoning, which corresponds to safety-critical scenarios identified in System-theoretic process analysis section . To test this, we quantify the level of agreement between complementary XAI methods and analyze how variations in consistency relate to potential interaction risks.

Within this framework, explanation consistency is operationalized as a safety-relevant signal that supports multiple design guidelines. In particular, it contributes to improving transparency of system behavior (G2), promoting calibrated trust (G4), and ensuring coherence across XAI outputs (G5). Furthermore, these consistency signals are intended to be integrated into the interaction layer of the system, where they can trigger adaptive feedback mechanisms such as clinician prompts and LLM-generated explanations to mitigate identified risks. Through this evaluation, we aim to demonstrate that explanation consistency is not merely a technical property of XAI methods, but a practical mechanism for detecting and managing safety risks in clinician-AI collaboration.

### Experimental setup

Experiments are conducted on the $PH^2$ dataset^26^^1^ which contains annotated images of nevus and melanoma. It is based on a dermoscopic image classification task. A total of 40 test images are used in the evaluation.

AlexNet^27^ is used as the base diagnostic model in this work. To support explainability, multiple XAI techniques are employed such as gradient-based attribution methods (e.g., Gradient-weighted Class Activation Mapping (Grad-CAM)^28^, integrated gradients^29^) and concept-based approaches. In particular, we use Testing with Concept Activation Vectors (TCAV)^30^ and extend concept-based analysis by projecting SHapley Additive exPlanations (SHAP)^31^ explanations onto the same concept space, enabling comparing between concept-level explanations across methods.

To assess the reliability of explanations, we analyze consistency across complementary XAI methods within two categories:

- Gradient-based attribution methods.
- Concept-based explanations.

This formulation enables a structured evaluation of explanation consistency across different interpretability modalities.

### Safety-oriented evaluation of explanation consistency

To operationalize the proposed safety framework, we quantify the level of agreement between complementary XAI methods and interpret this agreement as a proxy for the reliability and coherence of explanations. In contrast to traditional evaluations that assess explanation quality in isolation, we treat consistency between independent XAI methods as a safety-relevant signal, where disagreement may indicate ambiguity or instability in the model’s reasoning process.

We analyze consistency across two categories of explanation methods:

- *Gradient-based attribution methods*, which provide spatially localized importance maps.
- *Concept-based explanations*, which capture higher-level semantic contributions to model predictions.

For gradient-based explanations, both Grad-CAM and integrated gradients attribution maps are normalized to the range [0,1] and binarized using a fixed threshold prior to comparison. Agreement is then quantified using Intersection over Union (IoU)^32^, defined as the ratio between the intersection and union of the resulting salient regions. For concept-based explanations, local TCAV scores and SHAP concept importance values are first represented as vectors over the shared concept space. Agreement is computed using cosine similarity^33^, which measures the alignment between concept importance rankings.

For each input sample, pairwise similarity scores are computed within each modality. We report both the mean and variance of these scores across the test set to assess not only the overall level of agreement but also the stability of explanations across samples.

From a safety perspective, high agreement (agreement >= 0.5) between XAI methods suggests coherent and potentially more reliable explanations, supporting consistent interpretation by clinicians. Conversely, low agreement (agreement <= 0.4) indicates divergence in model reasoning, which may correspond to increased risk of misinterpretation or overconfidence in the system’s outputs.

The mean IoU across the test set was 0.13, indicating low spatial consistency between gradient-based methods. This suggests that attribution maps produced by Grad-CAM and integrated gradients often highlight different regions of the input image. However, substantial variability was observed, with a variance of 0.01, suggesting that the degree of agreement varies across samples. These findings highlight the sensitivity of spatial explanations to the choice of the XAI method and suggest potential ambiguity in how model reasoning is localized.

For concept-based explanations, the average cosine similarity was 0.70, indicating relatively higher consistency compared to gradient-based methods. Nevertheless, the observed variance of 0.15 suggests that the consistency score is not consistent across all samples. A subset of test samples exhibited lower similarity scores (below 0.50), revealing cases where concept importance rankings diverge across methods.

These low consistency cases represent situations where the model’s decision-making process may not be robustly captured by a single XAI method. From a safety standpoint, such disagreement can be interpreted as an indicator of increased uncertainty or complexity, warranting additional scrutiny during clinician-AI interaction.

Overall, these findings demonstrate that while a general level of agreement exists between XAI methods, inconsistencies are non-negligible and occur in a meaningful subset of cases. This supports the use of explanation consistency as a practical mechanism for identifying potentially unsafe or ambiguous scenarios, aligning with the safety design guidelines for promoting transparency (G2), calibrated trust (G4), and coherence (G5).

### Comparative study

To further investigate the safety implications of explanation consistency, we perform a comparative analysis across different XAI modalities. Rather than evaluating explanation methods in isolation, this analysis examines how differences in agreement between XAI methods relate to the reliability and interpretability of AI-generated diagnoses in clinician-AI interaction.

For gradient-based explanations, we compare attribution maps generated by Grad-CAM and integrated gradients using IoU. For concept-based explanations, we compare concept importance scores derived from TCAV and SHAP using cosine similarity.

This analysis allows us to assess:

1. The degree of agreement between independent XAI methods
2. The variability of explanations across test samples
3. The presence of significant divergence that may indicate potential safety risks

Gradient-based methods exhibited substantially lower agreement, with a mean IoU of 0.13, compared to concept-based explanations, which achieved a higher mean cosine similarity of 0.70. Although IoU exhibits a lower absolute variance compared to cosine similarity, its low mean value indicates consistently weak agreement between gradient-based XAI methods. In contrast, concept-based explanations achieved higher average agreement but with greater variability, suggesting that their reliability may vary across samples. From a safety perspective, both patterns highlight different forms of uncertainty that may impact clinician interpretation. While such diversity can offer richer insights, it may also introduce ambiguity when explanations conflict, potentially increasing the risk of misinterpretation.

To further examine these risks, we analyze test samples with low agreement between XAI methods, treating them as potential high-risk cases. Manual inspection of these low consistency cases revealed that disagreement often occurs in samples with ambiguous or complex visual patterns, where different methods focus on distinct regions or concepts. Such divergence reflects instability in how the model’s decision can be interpreted, which may not be apparent when relying on a single explanation method.

These findings reinforce the importance of explicitly analyzing agreement between XAI methods, as proposed in Guideline G5 (consistency and coherence of explanations). Moreover, the identification of low consistency cases provides a practical mechanism for flagging potentially unreliable or difficult to interpret predictions, supporting calibrated trust (G4) and improved transparency (G2). Overall, this comparative analysis demonstrates that explanation disagreement is not merely a methodological artifact, but a meaningful indicator of potential safety risks in clinician-AI interaction.

### Scenario-based safety validation

To assess the safety implications of the proposed framework, we perform a scenario-based validation derived from STPA. Rather than evaluating explanation methods in isolation, this analysis examines how explanation consistency can be used to detect and mitigate safety-critical interaction scenarios.

In addition, we consider a representative case in which a clinician evaluates an atypical nevus lesion in order to illustrate the practical application of the proposed framework. The CADx system predicts the image as melanoma with high confidence. However, the gradient-based consistency score remains relatively low (consistency score = 0.363). This discrepancy between the high confidence score and the low consistency score prompts the clinician to perform additional review rather than relying solely on the CADx recommendation. From an STPA perspective, this situation corresponds to risks associated with misleading or insufficiently coherent explanations (UCA-8) and inadequate verification of AI outputs (UCA-10). The proposed safety mechanisms, such as interactive clarification features (G3), reflection prompts (G4), and explanation consistency indicators (G5) are intended to mitigate these risks by encouraging critical assessment of the AI-generated diagnosis.

This representative case primarily illustrates the misinterpretation scenario. More generally, we consider two representative scenarios derived from the identified UCAs and loss scenarios:

- *Misinterpretation (UCA-8):* Explanations provided by the CADx system are unclear, incomplete, or conflicting, leading to incorrect understanding of the model’s decision.
- *Over-reliance/Automation bias (UCA-11):* Clinicians rely on AI outputs without verifying the underlying reasoning or considering alternative interpretations.

For each scenario, we analyze how inconsistencies between XAI methods manifest and how they can be leveraged as indicators of potential risk. Low agreement within each modality, is interpreted as a signal of ambiguity in the model’s reasoning process. For the purposes of this evaluation, we focus on misinterpretation of explanations and over-reliance/automation bias, as these are the hazards most directly addressed by explanation consistency signals.

For misinterpretation, inconsistencies across XAI outputs make conflicting reasoning explicit rather than implicit. This allows the system to highlight cases where different XAI methods emphasize distinct regions or concepts, thereby reducing the risk that clinicians form incorrect conclusions based on a single XAI method.

In the case of over-reliance/automation bias scenarios, the absence of explicit reliability indicators may lead clinicians to accept AI diagnoses without question. By providing a quantitative measure of explanation consistency, the system introduces an additional layer of transparency that supports more critical evaluation.

To operationalize these insights, the proposed framework integrates consistency signals into the interaction layer of the CADx system. When low agreement is detected, the system can trigger adaptive mitigation strategies, such as visual warnings, reflection prompts, or LLM-generated explanations that clarify the sources of disagreement between XAI methods. These mechanisms directly support the safety design guidelines by promoting interpretability (G1), calibrated trust (G4), and coherent presentation of explanations (G5).

Table 2 summarizes how explanation consistency contributes to detecting and mitigating risks across the considered scenarios. Overall, this analysis demonstrates that inconsistencies between XAI methods are not merely artifacts of different explanation techniques, but meaningful indicators of potential safety risks in clinician-AI interaction.

**Table 2:** Scenario based safety analysis illustrating how explanation consistency relates to potential interaction risks.

Scenario	Without consistency analysis	With consistency analysis
Misinterpretation	Conflicting XAI remains implicit	Inconsistency between XAI explicitly identified
Over-reliance/Automation bias	Clinician may accept AI recommendation without questioning supporting evidence	Low agreement between XAI reveals potential uncertainty and encourages additional verification before accepting the diagnosis

This analysis shows that inconsistencies between XAI methods can serve as indicators of potential risks in clinician-AI interaction. By making such inconsistencies explicit, the proposed approach provides additional signals that may support more informed interpretation of AI outputs and highlight cases requiring further scrutiny.

## Discussion

This study presents a systematic safety analysis of clinician-AI interactions in CADx systems, highlighting key socio-technical hazards such as automation bias, misinterpretation of XAI outputs, and cognitive overload. Using the STPA framework, we identified critical safety risks and derived a set of actionable safety design guidelines to address these challenges. These guidelines emphasize alignment between diagnostic outputs and XAI explanations, improved transparency of system behavior, and support for clinicians in maintaining situational awareness during diagnostic decision-making. Furthermore, each guideline is grounded in the identified UCA and loss scenario, ensuring traceability between safety analysis and design recommendations. Table 3 summarizes the traceability between identified UCAs, the derived safety design guidelines, and their implementation in the proposed GUI.

**Table 3:** Traceability between UCAs, safety design guidelines, and GUI features.

Guideline	Addressed UCAs	Safety rationale	GUI implementation
G1	UCA-7, UCA-8, UCA-9	Prevent misinterpretation of unclear or misleading XAI outputs	Structured explanation panels, simplified visualizations, LLM-generated summaries
G2	UCA-2, UCA-3, UCA-4	Avoid misinterpretation due to delayed outputs or unclear system confidence	Real-time status indicators, confidence score
G3	UCA-5, UCA-6	Enable clinicians to request clarification and avoid incomplete understanding	On-demand explanation buttons, adaptive prompts, interactive queries
G4	UCA-10, UCA-11, UCA-12	Prevent both over-reliance and under-reliance on AI-generated diagnoses	Reflection prompts, verification reminders, collaborative decision cues
G5	UCA-8	Avoid conflicting explanations that reduce trust	Agreement score across XAI models
G6	UCA-6, UCA-10	Reduce decision fatigue and improve usability in clinical workflows	Clean layout, progressive disclosure, integration of patient context

The results of the evaluation demonstrate that explanation consistency can serve as a meaningful indicator of reliability in AI-generated explanations. Across both gradient-based and concept-based methods, variability in agreement highlights that explanations are not always stable or aligned, particularly in complex or ambiguous cases. From a safety perspective, these inconsistencies are significant, as they correspond to scenarios identified in STPA where misinterpretation or over-reliance may occur.

The findings show that explanation disagreement is not merely a technical artifact of different XAI methods, but a signal that can reveal potential risks in clinician-AI interaction. Low consistency cases, in particular, indicate situations where the underlying model reasoning may be ambiguous or difficult to interpret, increasing the likelihood of incorrect conclusions if a single XAI is considered in isolation. This reinforces the importance of explicitly analyzing agreement between XAI methods, as reflected in Guideline G5, and supports the use of consistency as a mechanism for promoting calibrated trust (G4) and improved transparency (G2).

Nevertheless, explanation consistency alone should not be interpreted as a guarantee of safety. Agreement between multiple XAI methods provides only one perspective on explanation reliability and should be considered alongside other assurance mechanisms such as model robustness evaluation, performance validation, human oversight, and established safety engineering practices. In this work, consistency is treated as a complementary safety signal that may help identify situations requiring additional scrutiny rather than as a standalone measure of trustworthiness.

Although the proposed CADx interface incorporates LLM-generated summaries and explanation agreement scores to improve interpretability, these mechanisms do not inherently guarantee safe decision-making. For example, clinicians may over-rely on concise LLM-generated summaries without consulting the underlying explanations, potentially introducing automation bias. Similarly, presenting multiple indicators, such as confidence and agreement scores, may increase cognitive load if not designed appropriately. In the proposed framework, these elements are intended to support reflective decision-making rather than replace clinician judgment. Their effectiveness and usability will be investigated through future clinician-centered user studies.

More broadly, this work demonstrates that safety in CADx systems extends beyond predictive accuracy to include the interpretability and coherence of explanations presented to clinicians. By linking STPA-derived hazards to measurable properties of XAI outputs, the proposed framework provides a structured approach for identifying and mitigating risks in clinician-AI collaboration. This highlights the value of integrating safety analysis with explainability techniques, enabling the design of systems that not only perform well but also support safe and informed decision-making.

While this work focuses primarily on clinician-AI interaction and interface-level considerations, other factors identified in the analysis, including algorithmic limitations and system-level constraints, remain important for achieving comprehensive CADx system safety. Alignment with established frameworks such as FUTURE-AI and AAMI/BSI can further support robustness, reliability, and governance across the system lifecycle.

Despite offering a structured and systematic approach, this study has several limitations. First, the current evaluation is based on simulated scenarios and retrospective analysis of explanation consistency, rather than real-world clinician interaction. Second, while the proposed CADx interface implements multiple safety-oriented interaction mechanisms, including LLM-generated summaries and explanation consistency indicators, the impact of these features on clinician trust calibration and cognitive workload has not yet been empirically evaluated. Empirical validation through user studies and clinical evaluations is needed to assess how clinicians interpret and respond to consistency signals in practice. Furthermore, explanation consistency itself has inherent limitations as a safety indicator. Multiple XAI methods may produce highly consistent explanations while collectively reflecting the same incorrect or biased model behavior. Also, disagreement between explanations does not necessarily imply that the underlying diagnosis is erroneous, as different methods may capture different aspects of the model’s reasoning process. Third, the focus on dermoscopic CADx systems may limit generalizability to other clinical domains, such as treatment planning or prognostic modeling, where different interaction risks may arise. Finally, applying STPA in this context requires detailed knowledge of both system behavior and clinical workflows, which may vary across healthcare settings.

## Conclusion and future work

In conclusion, ensuring the safety of clinician-AI interaction is essential for the responsible deployment of CADx systems in clinical practice. In this work, we presented a systematic and human-centered approach to analyzing and mitigating risks in clinical-AI collaboration, grounded in the STPA framework. By explicitly modeling socio-technical hazards, we derived a set of actionable safety design guidelines to support safer and more effective clinical decision-making.

To bridge the gap between safety analysis and system design, these guidelines were operationalized within a CADx interaction framework, demonstrating how safety considerations can be embedded directly into the interface and interaction layer. In addition, we introduced a safety-oriented evaluation approach, showing that consistency across multiple XAI methods can serve as a quantitative signal for identifying potentially unreliable or ambiguous explanations. This denotes that explanation consistency is not only a technical property of interpretability method, but a practical mechanism for detecting and managing safety risks in clinician-AI interaction.

The findings highlight that achieving safety in CADx systems extends beyond predictive performance to include clarity, coherence, and reliability of explanations provided to clinicians. By linking STPA-derived risks to measurable properties of XAI outputs, this work provides a structured foundation for designing AI systems that support calibrated trust and informed decision-making.

Future research will focus on validating the proposed framework through user-centered studies and real clinical evaluations, to investigate how clinicians interpret and respond to explanation consistency signals and adaptive feedback mechanisms. Furthermore, extending this approach to other clinical AI applications and integrating it with broader safety and governance frameworks will be important for advancing trustworthy and human-centered AI in healthcare. Overall, this work contributes toward the development of CADx systems that function not only as accurate diagnostic tools, but as safe, transparent, and reliable partners in clinical decision-making.

## Data Availability

All data generated or analyzed during this study are included in this published article: “T. Mendonça, P. M. Ferreira, J. S. Marques, A. R. S. Marcal and J. Rozeira, “PH2 - A dermoscopic image database for research and benchmarking,” 2013 35th Annual International Conference of the IEEE Engineering in Medicine and Biology Society (EMBC), Osaka, Japan, 2013, pp. 5437-5440, doi: 10.1109/EMBC.2013.6610779”.

## Appendix A: Main responsibilities of the clinician, CADx system, and explainable module in the CADx system

The main responsibilities as seen in Fig. 2 are summarized as follows:

1. Clinician (human controller)
  - R1 *Executes CADx system*: Initiates the diagnostic process by inputting relevant patient data.
  - R2 *Requests clarification or feedback from CADx system*: Queries the CADx system for additional insights or explanations if needed [SC-2, SC-4].
  - R3 *Verifies diagnosis by CADx system*: Cross-checks the CADx’s output with clinical expertise [SC-1, SC-2, SC-5].
  - R4 *Provides diagnosis based on the output of CADx system*: Combines the CADx outputs with clinical judgment to arrive at a final diagnosis [SC-1, SC-5].
2. CADx system (automated controller)
  - R5 *Provides diagnosis*: Analyzes input data and outputs a diagnosis [SC-3, SC-5].
3. Explainable module in the CADx system (automated controller)
  - R6 *Communicates with CADx system*: Processes and provides understandable explanations or justifications for the diagnosis when requested by the clinician [SC-3, SC-4, SC-5].

## Appendix B: Full list of identified UCAs

The full list of identified UCAs can be seen in Table 4.

## Appendix C: Full list of derived CCs

The full list of derived CCs based on their corresponding UCAs are as follows:

- CC-1: CADx system must provide a preliminary diagnosis output [UCA-1].
- CC-2: CADx system must not provide a preliminary diagnosis output that is incorrect or misleading [UCA-2].
- CC-3: CADx system must not provide a preliminary diagnosis output too late [UCA-3].
- CC-4: CADx system must not halt its process prematurely before providing a diagnosis output [UCA-4].
- CC-5: Clinician must request for clarification or additional information from CADx system [UCA-5].
- CC-6: Clinician must not request clarification or additional information repeatedly [UCA-6].
- CC-7: Explainable module must provide explanation of diagnosis rationale [UCA-7].
- CC-8: Explainable module must not provide explanation of diagnosis rationale that is incomplete, misleading, or not understandable by clinician [UCA-8].
- CC-9: Explainable module must not provide explanation of diagnosis rationale too late [UCA-9].
- CC-10: Clinician must review and verify diagnosis output by CADx system [UCA-10].
- CC-11: Clinician must not review and verify diagnosis output by CADx system wrongly [UCA-11].
- CC-12: Clinician must make final diagnosis decision based on CADx system output and personal clinical judgment [UCA-12].

**Table 4:** Identified unsafe control actions.

Control actions	Not provided	Provided	Too early, too late	Stopped too soon, applied too long
CADx system provides preliminary diagnosis output	UCA-1: CADx system *does not provide* a preliminary diagnosis output [H-4, H-5]	UCA-2: CADx system *provides* a preliminary output, but it is incorrect or misleading [H-1, H-3, H-5]	UCA-3: CADx system *provides* a preliminary output too late [H-4, H-5]	UCA-4: CADx system halts its process *prematurely* [H-4, H-5]
Clinician requests clarification or additional information	UCA-5: Clinician *does not request* for clarification or additional information [H-2, H-4, H-5]	UCA-6: Clinician *requests* for clarification or additional information repeatedly [H-1, H-4, H-5]	N/A	N/A
Explainable module provides explanation of diagnosis rationale	UCA-7: Explainable module *does not provide* explanation of diagnosis rationale [H-4, H-5]	UCA-8: Explainable module *provides* explanation of diagnosis rationale, but it is incomplete, misleading, or not understandable by clinician [H-3, H-4, H-5]	N/A	UCA-9: Explainable module provides explanation of diagnosis rationale *too late*, after clinician has already made a diagnostic decision [H-4]
Clinician reviews and verifies diagnosis output	UCA-10: Clinician *does not review* or verify diagnosis output [H-2]	UCA-11: Clinician *reviews* and verifies diagnosis output, but it is wrong [H-1]	N/A	N/A
Clinician makes final diagnosis decision	N/A	UCA-12: Clinician *makes* final diagnosis decision without considering CADx system output [H-2]	N/A	N/A

## Appendix D: Full list of loss scenarios

The full list of loss scenarios are identified as follows:

- *Scenario-1 for UCA-1*: CADx system does not provide a preliminary diagnosis output due to a software malfunction, causing delays and requiring the clinician to rely on less accurate or timely information.
- *Scenario-2 for UCA-1*: CADx system does not provide a preliminary diagnosis output because the clinician did not execute it, possibly due to algorithm aversion.
- *Scenario-3 for UCA-2*: CADx system provides a preliminary output, but it is incorrect or misleading due to flaws in the AI algorithms, resulting in an incorrect or misleading output.
- *Scenario-4 for UCA-2*: CADx system provides a preliminary output, but it is incorrect or misleading due to flaws in the AI algorithms. This misalignment between the clinician’s expectations and the CADx system’s predictive capabilities may lead to over-reliance or misinterpretation of the output provided.
- *Scenario-5 for UCA-3*: Due to resource limitations e.g., insufficient computational resources, the preliminary output is delayed within the CADx system, which prevents the timely update of the GUI, disrupting the diagnosis workflow and causing potential misunderstandings.
- *Scenario-6 for UCA-3*: CADx system incurs delays due to flaws in the AI algorithm, leading to a gap in expected response time, causing the clinician to assume an error, which disrupts their reliance on the system.
- *Scenario-7 for UCA-4*: CADx system halts its process prematurely due to resource limitations e.g., insufficient computational resources, leading to a loss of clinician trust and increased likelihood of algorithm aversion.
- *Scenario-8 for UCA-4*: CADx system halts its process prematurely due to an unanticipated software malfunction, reducing clinician trust in the system.
- *Scenario-9 for UCA-5*: Clinician does not request for clarification or additional information due to over-reliance on the CADx diagnosis, which discourages further verification or clarification.
- *Scenario-10 for UCA-5*: Clinician does not request for clarification or additional information due to algorithm aversion.
- *Scenario-11 for UCA-5*: Clinician does not request for clarification or additional information due to a lack of clear prompts from the CADx system about its uncertainties or limitations, preventing the clinician from recognizing the need for further clarification.
- *Scenario-12 for UCA-6*: Clinician requests for clarification or additional information repeatedly due to incorrect or inappropriate explanations from the explainable module. As a result, the clinician’s expectation and trust in the system declines over time.
- *Scenario-13 for UCA-6*: Clinician requests for clarification or additional information repeatedly due to interface design shortcomings, impacting the clinician’s ability to comprehend explanations.
- *Scenario-14 for UCA-6*: Clinician requests for clarification or additional information repeatedly due to CADx system’s failure to output a diagnosis. As a result, the clinician’s expectation and trust in the system declines over time.
- *Scenario-15 for UCA-7*: Explainable module does not provide explanation of diagnosis rationale due to flaws in the AI algorithms. As a result, the clinician’s expectation and trust in the system declines over time.
- *Scenario-16 for UCA-7*: Explainable module does not provide explanation of diagnosis rationale due to the clinician failing to request an explanation from the explainable module, possibly as a result of mistrust in the system.
- *Scenario-17 for UCA-7*: Explainable module does not provide explanation of diagnosis rationale due to missed or delayed requests from the clinician, possibly due to unanticipated software malfunction. As a result, there is a miscommunication between the clinician and the explainable module.
- *Scenario-18 for UCA-8*: Explainable module provides an explanation of the diagnosis rationale, but it is incomplete, misleading, or not understandable by clinician due to flaws in the AI algorithms, leading to clinician misinterpretation of AI reasoning and potentially result in incorrect diagnostic decision.
- *Scenario-19 for UCA-9*: Explainable module provides an explanation of the diagnosis rationale too late, after clinician has already made a diagnostic decision due to flaws in the AI algorithms, resulting in missed or delayed feedback.
- *Scenario-20 for UCA-10*: Clinician does not review or verify diagnosis output due to algorithm aversion bias, possibly stemming from prior errors or a lack of understandable explanations, leading the clinician to skip necessary verification and potentially compromising patient safety.
- *Scenario-21 for UCA-11*: Clinician reviews and verifies diagnosis output, but it is wrong due to automation bias, leading the clinician to trust the system output without thorough verification, potentially resulting in incorrect conclusions if the CADx diagnosis is inaccurate.
- *Scenario-22 for UCA-12*: Clinician makes a final diagnosis decision without considering the CADx system output due to prior negative experiences, unclear explanations, or other factors that increase algorithm aversion, leading the clinician to exclude the CADx output from the final decision-making process.

## Appendix E: Full list of detailed safety goals, safety requirements, and mitigation strategies

This list provides the detailed derivation of the proposed safety design guidelines presented in the main paper. It outlines the intermediate SGs, SRs, and MSs identified through STPA. These elements were subsequently consolidated and abstracted into a set of actionable safety design guidelines for clinician-AI interaction in CADx systems. The derivation of safety design guidelines are as follows:

- *SG Category (3) - GUI design gaps*: These goals aim to ensure that the CADx interface supports clarity, transparency, and cognitive efficiency in clinician-AI interaction.
  - SG1: Ensure clarity of diagnostic information from the different XAI models.
    - SR1: Explanations shall be accessible and contextually relevant to support clinicians in the decision-making process.
    - MS1: Propose regular validation of XAI models with clinical end users to ensure that clarity of the information in the GUI improves over time.
  - SG2: Enhance transparency of CADx system status and reliability.
    - SR2: CADx system status updates, such as real-time processing indicators or error alerts, shall be displayed to inform clinicians if a diagnosis is delayed, incomplete, or requires re-evaluation.
    - MS2: Implement real-time alert notifications and visual cues regarding system status within the GUI.
  - SG3: Support clinician decision-making with clear explanation prompt.
    - SR3: A prompt system shall be integrated within the GUI to notify clinicians of any limitations or uncertainties in the CADx diagnosis, along with options for requesting further clarification.
    - MS3: Integrate adaptive prompts that can change based on the clinician preferences.
  - SG4: Facilitate trust and avoid over-reliance on CADx output.
    - SR4: The clinician judgment shall play a key role in the final decision, with the CADx system serving as a supportive resource.
    - MS4: Implement a collaborative reminder system within the GUI to emphasize the importance of clinician expertise in conjunction with CADx system insights.
  - SG5: Minimize cognitive load and decision fatigue.
    - SR5: GUI design standards shall be adhered to in order to provide clear, structured options for requesting explanations and to minimize unnecessary visual clutter.
    - MS5: Use of usability testing protocols to validate the interface design of the GUI with clinicians.
- *SG Category (4) - Clinicians’ trust*: These goals aim to ensure that clinicians develop and maintain appropriate, well-calibrated trust in the CADx system through transparent, multi-source explanations, and clear communication of diagnostic reliability.
  - SG6: Strengthen trust through multi-explanation consistency and transparency.
    - SR6: The CADx diagnostic outputs shall align with insights from multiple XAI modules, with discrepancies clearly indicated to clinicians.
    - MS6: Display explanations from multiple XAI modules together with an agreement score quantifying their consistency.
  - SG7: Support interpretability through contextualized summary explanations.
    - SR7: A summary report shall be automatically generated by an integrated Large Language Model (LLM) to synthesize diagnostic reasoning from multiple XAI outputs.
    - MS7: Provide an editable, LLM-generated explanation panel that allows clinicians to review, question, or annotate the AI’s reasoning for auditability and trust calibration.
  - SG8: Encourage proper usage of CADx system.
    - SR8: Proper training materials or built-in tooltips shall be implemented to help clinicians understand the CADx system’s intended use and limitations, discouraging both over-reliance and under-reliance.
    - MS8: Provide an interactive simulation-based training to familiarize clinicians with CADx system.
  - SG9: Foster confidence by displaying the CADx system’s diagnostic confidence score and agreement scores of XAI models.
    - SR9: Confidence scores for CADx outputs shall be displayed alongside diagnostic explanations, ensuring that clinicians can interpret the CADx predictions in the context of their clinical expertise.
    - MS9: Develop a “second opinion” feature that allows clinicians to compare CADx outputs with colleagues.
  - SG10: Avoid automation bias.
    - SR10: Reminders shall be integrated within the GUI that encourage clinicians to work hand-in-hand with the CADx system, reinforcing collaborative decision-making and reducing over-reliance on the system’s diagnosis.
    - MS10: Include a “reflection” feature within the GUI, prompting clinicians to review key decisions points influenced by the CADx system.

## Footnotes

1 PH2 Dataset: https://www.fc.up.pt/addi/ph2%20database.html,
